# Clival chordoma: long-term clinical outcome in a single center

**DOI:** 10.1097/MD.0000000000012207

**Published:** 2018-09-07

**Authors:** Yibiao Zhou, Bolin Hu, Zhiwei Wu, Hanxiong Cheng, Min Dai, Bin Zhang

**Affiliations:** Department of Orthopedics, The First Affiliated Hospital of Nanchang University, Nanchang, Jiangxi, China.

**Keywords:** chemotherapy, clival chordoma, gamma knife radiosurgery, maximally safe cyto-reductive resection, multimodal treatment, radiosurgery

## Abstract

The treatment of clival chordoma remains highly challenging. This difficulty is enhanced by the very small likelihood of a successful complete surgical resection or nonsurgical treatment of chordoma. Additionally, no effective means of interdisciplinary treatment for chordoma have been identified. With this background, we analyzed data of patients who underwent multidisciplinary treatment for clival chordoma at our institution during the last 25 years.

This retrospective study evaluated patients treated at a single center from 1992 to 2017.

During the study period, 24 patients underwent 24 surgeries. Twenty-two surgical resections (including 1 initial surgery and 1 surgery for recurrence) were deemed maximally safe cyto-reductive resections (92%); the remaining 2 surgeries were deemed incomplete (8%), which were histologically confirmed in all but in 1 case (which involved radionecrosis). The complications were divided into endocrinologic, neurologic, and other complications. In 1 case (4%), surgery led to immediate dyspnea followed by death on the following day; in another case (4%), ischemic infarction led to sudden death. In 3 cases (13%), patients exhibited improvements of neurologic (visual or oculomotor) deficits that had been observed prior to surgery. The following new postoperative neurologic deficits were observed: oculomotor deficits in 4 cases, dizziness in 2 cases, and cranial nerve-attributed dysphagia in 3 cases. About 19 patients underwent adjuvant postoperative radiotherapy following the initial surgery (dose: 54.5 Gy in all cases). The mean and median follow-up durations were 50 ± 53 and 48.5 months, respectively. A Kaplan–Meier analysis estimated a median survival duration of 50.2 months (95% confidence interval 27.9–72.4 months).

These findings highlight the importance of interdisciplinary treatment strategies, particularly those combining maximally safe cyto-reductive tumor resection and adjusted radiotherapy and other treatment options, for patients with relatively good conditions.

## 1 Introduction

The reported incidence of clival chordoma arising from the primitive notochord remnant is approximately 8 cases per 10 million people.^[1]^ Recently, Walcott et al published an overview of clival chordoma and the currently available treatment strategies.^[2]^ However, the lack of evidence present challenges to the study of surgical resection and subsequent proton beam therapy for clival chordomas, for which gross total resection is often impossible owing to the involvement of vulnerable neurovascular structures and the diffusely infiltrative growth pattern of these lesions. Treatment options for clival chordoma and hypotheses of prospective trials will likely benefit from better clinical data, including the nuances and details of tumor outcomes, as well as reports of the chronic aspects of the disease.

With this study, we aimed to meet the need for such data. We analyzed all patients diagnosed with clival chordoma at our institution between 1992 and 2017. This period is characterized by a neurosurgical paradigm that entailed maximally safe cyto-reductive resection with the intent to avoid neurologic deficits, followed by proton beam therapy.

## 2 Methods

This retrospective clinical study was approved by the board of the local ethics committee. All patients surgically treated for histologically proven chordoma at our institution between 1992 and 2017 were included. Data were obtained from patient files, including the institutional radiologic imaging database; follow-up data were obtained entirely by standardized telephone interviews. All survivors completed the Short Form-36-Health Survey (SF36) at the time of data acquisition.

All patients underwent surgical treatment via standard and skull base approaches. A combined surgical approach was defined as the performance of a second procedure within 12 weeks after the initial surgery. These combined procedures were considered a single surgery for analytical purposes.

The follow-up evaluations comprised a clinical examination and magnetic resonance imaging scan and were usually conducted at 3 and 12 months postoperatively and every 12 months thereafter. Recurrence was considered in cases involving any radiologically suspected new tumor expansion after maximally safe cyto-reductive resection or known residual tumor after an incomplete resection.

The statistical analysis was performed using IBM SPSS Statistics 22 (IBM SPSS, North Castle, NY). The cumulative and progression-free survival rates were estimated using a Kaplan–Meier analysis. The SF36 questionnaire data were analyzed using Excel (Microsoft Corp, Redmond, WA). Interval-scaled data are expressed as mean or median with respective deviations, and nominal data are expressed as absolute numbers and valid percentages.

## 3 Results

### 3.1 Clinical features

Twenty-four patients (13 men, 11 women) were treated for cranial chordoma at our institution during the study period; these patients underwent a total of 24 surgical procedures. The mean age at diagnosis was 42 ± 14 years. Of the 24 patients, 1 only underwent a minimally invasive transoral biopsy at our institution, while all subsequent treatment and follow-up information was obtained through our work.

One patient was lost to follow-up. The symptoms that led to diagnosis were dysphagia in 5 patients (21%), double vision in 15 patients (63%), and headache and other nonspecific signs in 6 patients (23%) (Table 1).

**Table 1:**
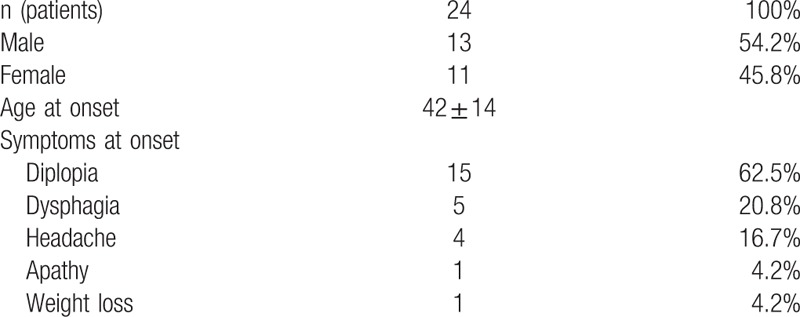
Patient collective and clinical features.

### 3.2 Surgical

Twenty-two surgical resections (including 1 initial surgery and 1 surgery for recurrence) were deemed maximally safe cyto-reductive resections (92%); the remaining 2 surgeries were deemed incomplete (8%). Two patients underwent re-operation up to 4 times for radiologic recurrences of chordoma, which were histologically confirmed in all but in 1 case (which involved radionecrosis). Altogether, the 24 patients underwent a total of 24 surgeries. As none involved a combined approach, this total represented 24 individual surgical procedures. Table 2 lists the various surgical approaches according to the location of the clival chordoma.

**Table 2:**
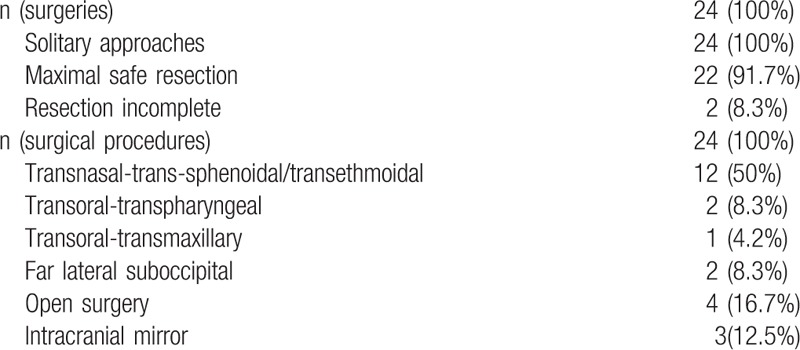
Analysis of surgical procedures applied for chordoma resection.

The complications were divided into endocrinologic, neurologic, and other complications (Table 3). In 1 case (4%), surgery led to immediate dyspnea followed by death on the following day; in another case (4%), ischemic infarction led to sudden death. In 3 cases (13%), patients exhibited improvements of neurologic (visual or oculomotor) deficits that had been observed prior to surgery.

**Table 3:**
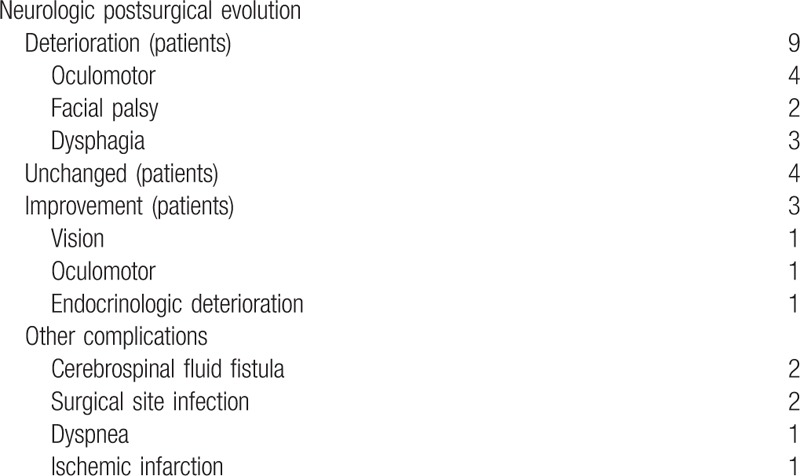
Neurologic, endocrinologic, and other complications following chordoma resection.

The following new postoperative neurologic deficits were observed: oculomotor deficits in 4 cases, dizziness in 2 cases, and cranial nerve-attributed dysphagia in 3 cases (none requiring tracheostomy or long-term feeding tubes). Furthermore, nonneurologic postoperative complications were found in 5 cases: surgical site infection in 2 cases, cerebrospinal fluid fistulae in 2 cases, and dysphagia leading to death in 1 case.

### 3.3 Histology and further treatment strategies

In all cases, diagnoses of chordoma were confirmed by histopathology, with no or very low mitotic activity apparent in hematoxylin and eosin-stained sections or a proliferation index of 1 ± 5% as estimated by anti-Ki67 (MIB1) immunostaining.

### 3.4 Radiotherapy

About 19 patients underwent adjuvant postoperative radiotherapy following the initial surgery (dose: 54.5 Gy in all cases). Eighteen patients underwent proton beam therapy and 1 underwent 12 sessions of gamma knife therapy (Elekta Instrument AB, Stockholm, Sweden). All proton beam treatments were administered at the same center, with a median dose of 54.5 Gy relative biologic effectiveness (RBE) in daily fractions of 2.0 Gy RBE. An RBE factor for protons of 1.1 (relative to that of 60 Co) was used. Patients were treated on the institute's 2 scanning gantries using the pencil beam scanning technique and energy-degraded beams from 590- or 250-MeV-dedicated medical cyclotrons. Complications from radiotherapy (other than moderate asthenia, alopecia, moderate mucosal complications) included unilateral useful-hearing loss in 2 patients, vision loss of <50% in 1 patient, and oculomotor deficits in 1 patient.

### 3.5 Outcomes

The mean and median follow-up durations were 50 ± 53 and 48.5 months, respectively. One patient was lost to all follow-up. Seven patients died before the end of the data acquisition period, and all 7 deaths were attributed to chordoma (overall mortality rate, 29%). The remaining 16 patients had no major deficits (mean Karnofsky Performance Score [KPS], 90%; range 70–100%). The 5-year cumulative cause-specific survival rate was 46% (95% confidence interval [CI] 54–96%), and the 5-year progression-free survival rate was 33% (95% CI 23–69%). The mean cause-specific survival and progression-free survival durations were 99.9 ± 47.2 and 100.4 ± 53.4 months, respectively, and 8 patients remained alive at the end of the data acquisition period (Table 4). Therefore, the mean survival durations are considered intermediate and will become more accurate over time.

**Table 4:**
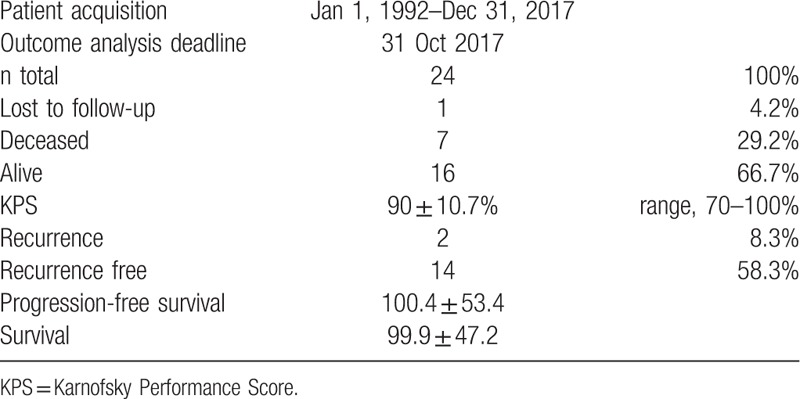
Long-term outcome after multimodal treatment.

**Figure 1:**
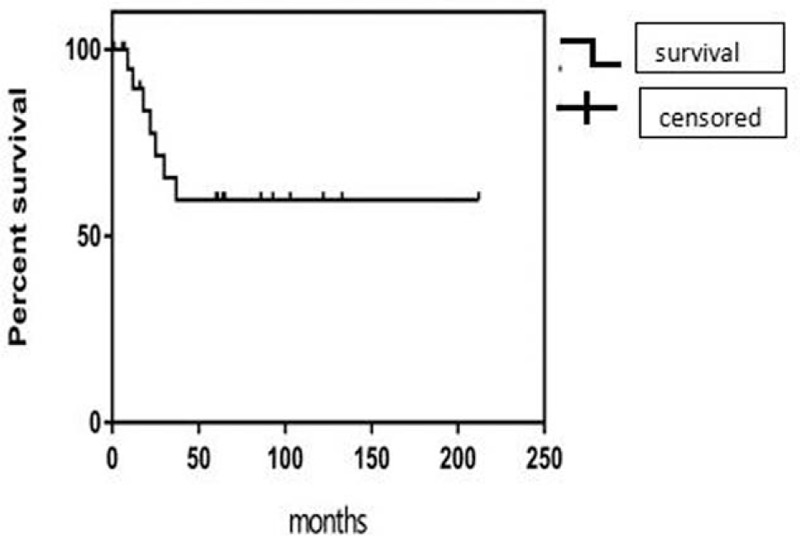
The Kaplan–Meier analysis of survival after diagnosis. A Kaplan–Meier analysis estimated a median survival duration of 50.2 months (95% confidence interval 27.9–72.4 months). Sixteen of 24 patients remained alive and assessable during follow-up, yielding an overall survival rate of 66.7%. Seven of 24 patients died during follow-up, and tumor progression was considered the cause of death in all such cases. Additionally, 1 patient was lost to follow-up.

All 16 survivors had completed the SF36 Questionnaire at the time of data acquisition (Fig. 1). The summed scores of the Physical and Mental Component components were 43 (13% above, 37% at, and 50% below the general population norm) and 47 points (25% above, 38% at, and 38% below the general population norm).

**Figure 2:**
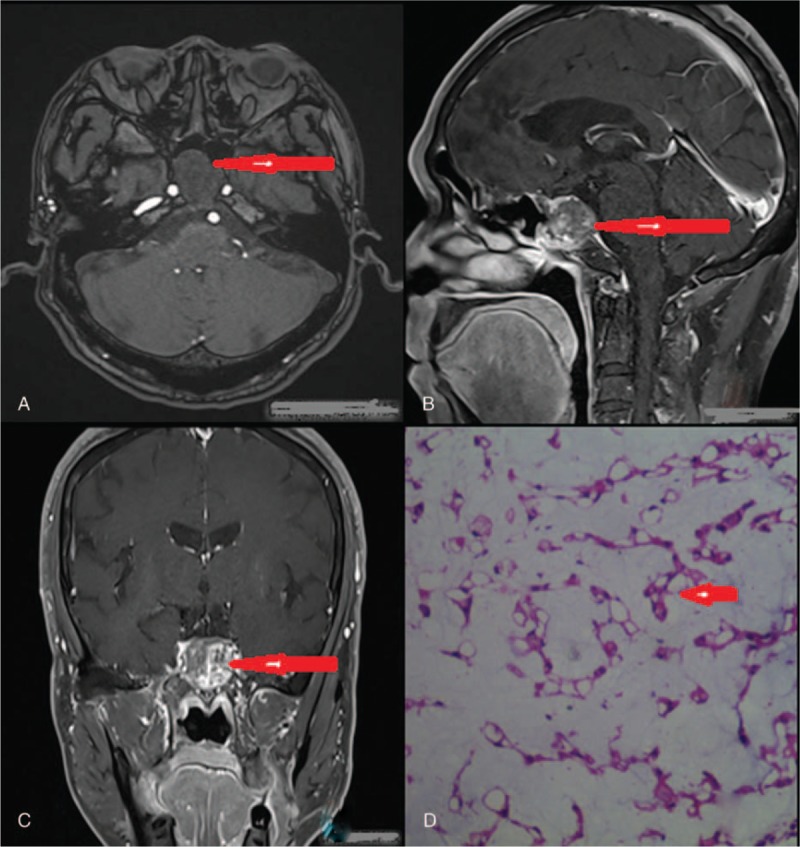
The imaging and histologic characteristics of typical case. (A–C) Magnetic resonance imaging showed that the solid occupying lesions are seen in the saddle-bottom slope area. The enhanced scan shows uneven and moderate enhancement, and the pituitary pressure rises (as arrow heads). (D) Histologic sections of the mass displayed tumor tissue in a myxoid background and cords and lobules of vacuolated physaliphorous cells with abundant cytoplasm and a large amount of mucus. The nucleus was round or oval without definite mitosis (original magnification, ×100) (as arrow heads).

A Kaplan–Meier analysis estimated a median survival duration of 50.2 months (95% CI 27.9–72.4 months). Sixteen of 24 patients remained alive and assessable during follow-up, yielding an overall survival rate of 66.7%. Seven of 24 patients died during follow-up, and tumor progression was considered the cause of death in all such cases. Additionally, 1 patient was lost to follow-up (patient 3) (Table 5 and Fig. 2).

**Table 5:**
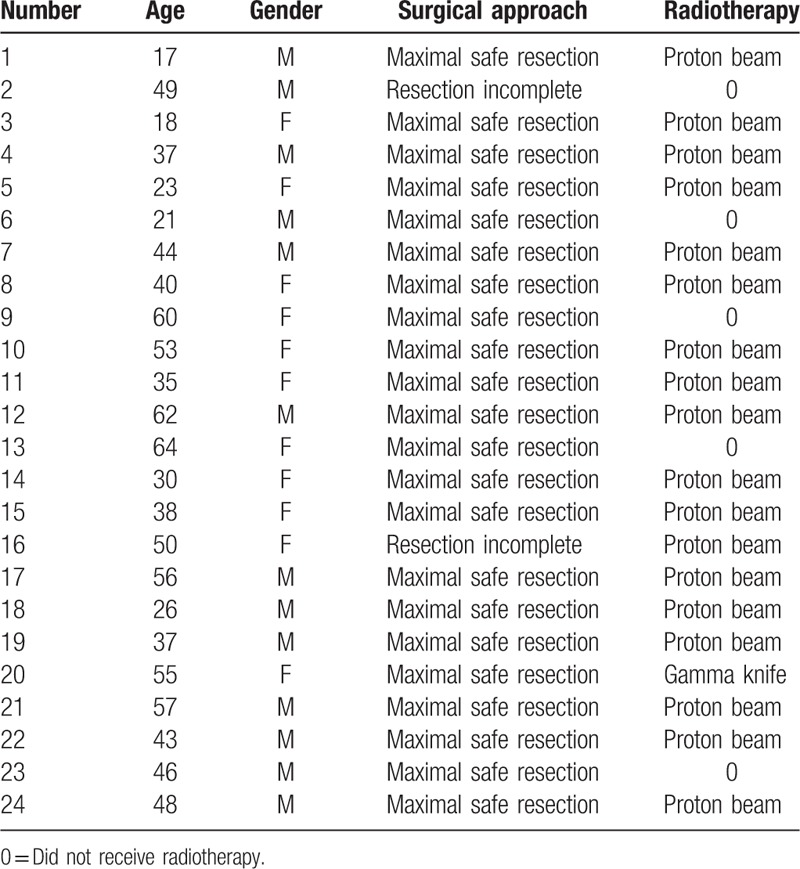
Information about the individual therapy for each patient.

## 4 Discussion

Our data provide important information about the diagnosis and treatment of clival chordoma.

In a meta-analysis of 23 publications, Di Maio et al^[3]^ determined an overall 5-year survival rate of 70% to 78%. Our rate of 73%, therefore, was consistent with that reported in the meta-analysis. Furthermore, the meta-analysis determined a progression-free survival rate of 54% and consistent with our rate of 55%. As functional outcomes were not reported in the meta-analysis, our patient-subjective (SF36) and physician-estimated (KPS) outcomes among the survivors can be compared to single studies.^[1,4–11]^

The surgical approaches and neurologic and nonneurologic complications in our study were consistent with those in other studies.^[4,5,9,11,12]^ However, Di Maio et al reported that the rates of maximally safe cyto-reductive resection ranged from 0% to 74%^[3]^; therefore, our rate of 92% could be considered high. Despite sufficient evidence of potential efficacy for sacral chordoma,^[13]^ this treatment concept cannot be simply transferred to the treatment of clival chordoma, which are rarely indicated for en bloc resection and gross total tumor resection. Additionally, the publication bias observed in chordoma studies is usually influenced by the small numbers of patients.

The comparison of a maximally safe resection with the theoretical oncologic advantage of total structural resection may be more valid.^[11–15]^ The latter procedure may not be virtually complete or may be achieved at the expense of postoperative deficits that contribute to morbidity and delays in adjuvant therapy in some cases.^[2,3,7,11]^

In this study, the good postoperative KPS scores of patients reflected the often mild and temporary nature of surgical complications with regard to the long-term follow-up and indicated that most survivors were able to perform daily activities with no or minor symptoms of disease (mean KPS, 90%; worst KPS, 70%).

As 19 of 24 patients underwent adjuvant radiotherapy following the initial surgery, it is not ideal to conduct separate analyses of the effects of surgery and radiotherapy on tumor control. Most patients received proton beam therapy, which delivers high doses of radiation while sparing adjacent neural structures and is thus recommended for chordoma; however, no studies have proven the superiority of proton beam relative to other therapies.^[16]^ The complications of radiotherapy observed in this study were consistent with those published in some previous studies.^[2,7,8,14,15]^

In our study, we observed the following issues related to treatment. First, diagnosis was not achieved in a timely manner; once a chordoma was identified, it usually had become quite large in volume and had compressed important surrounding neural tissues, leading to serious clinical symptoms. Therefore, we were unable to achieve complete tumor resection, and radiotherapy protocols were performed while considering the protection of the surrounding vital nerve tissues. Second, surgery is considered the first line of treatment for chordoma, and most of our cases involved surgical treatment. However, 1 patient did not undergo surgical treatment but rather was subjected to diagnostic imaging and pathology, followed by 12 sessions of gamma knife treatment, which has been clinically effective. To date, relatively few gamma knife treatments have been performed, although the therapeutic effect has been confirmed by relevant studies.^[17]^ Therefore, additional relevant studies should be conducted under appropriate conditions to provide information and evidence regarding more effective methods for the treatment of chordoma. Third, our follow-up study could not achieve satisfactory results, monitor the patients’ treatment courses and cause partial patients to lose their visit, and further lose the number of samples that are already small.

### 4.1 Limitations of the study

This was a retrospective study, and the lack of standardized treatment and small series have invalidated many statistical comparisons and evaluations. Our data reflect the experiences and outcomes of chordoma patients treated at a nonsubspecialty center. This type of bone cancer is so rare that most single-center analyses will remain descriptive.

## 5 Conclusion

We endorse the concept of maximally safe surgical resection rather than total resection for cranial chordomas. High-profile interdisciplinary tumor boards may compensate for differences in caseloads among average-sized neurosurgical centers. Our findings confirm those of previous reports in the literature that indicate that clival chordomas should be considered a chronic progressive disease that requires continuous monitoring and treatment of potential recurrences, irrespective of adjuvant radiotherapy. It is, therefore, neither realistic nor rational to plan for a 1-time cure comprising maximally safe surgery and adjuvant radiotherapy.^[18–20]^

## Author contributions

ZYB conceived and conducted the experiments and prepared the manuscript. HBL contributed to data sorting and analysis. WZW and CHX helped with the analysis and constructive discussions. ZB and DM contributed to the conception of the study and approved the final manuscript. All authors read and approved the final manuscript.

**Data curation:** Yibiao Zhou, Bolin Hu, Min Dai, Bin Zhang.

**Funding acquisition:** Yibiao Zhou, Bolin Hu, Bin Zhang.

**Investigation:** Yibiao Zhou, Bolin Hu, Zhiwei Wu, Hanxiong Cheng.

**Methodology:** Yibiao Zhou, Bolin Hu, Zhiwei Wu, Hanxiong Cheng.

**Project administration:** Yibiao Zhou, Bolin Hu, Zhiwei Wu, Hanxiong Cheng.

**Resources:** Yibiao Zhou, Hanxiong Cheng.

**Formal analysis:** Bolin Hu, Min Dai, Bin Zhang.

**Conceptualization:** Min Dai, Bin Zhang.
